# Non-Targeted Spectranomics for the Early Detection of *Xylella fastidiosa* Infection in Asymptomatic Olive Trees, cv. Cellina di Nardò

**DOI:** 10.3390/molecules28227512

**Published:** 2023-11-09

**Authors:** Elhussein Ahmed, Biagia Musio, Stefano Todisco, Piero Mastrorilli, Vito Gallo, Maria Saponari, Franco Nigro, Stefania Gualano, Franco Santoro

**Affiliations:** 1 Department of Civil, Environmental, Land, Building Engineering and Chemistry (DICATECh), Polytechnic University of Bari, Via Orabona, 4, I-70125 Bari, Italy; elhussein.ahmed@poliba.it (E.A.); stefano.todisco@poliba.it (S.T.); piero.mastrorilli@poliba.it (P.M.); vito.gallo@poliba.it (V.G.); 2 International Centre for Advanced Mediterranean Agronomic Studies of Bari (CIHEAM Bari), Via Ceglie 9, 70010 Valenzano, Italy; fsantoro@iamb.it; 3 Innovative Solutions S.r.l.—Spin-Off Company of Polytechnic University of Bari, Zona H 150/B, 70015 Noci, Italy; 4 Istituto Per la Protezione Sostenibile Delle Piante, CNR, Via Amendola 122/D, I-70126 Bari, Italy; maria.saponari@cnr.it; 5 Department of Soil, Plant and Food Sciences, University of Bari Aldo Moro, Via Orabona, 4, I-70125 Bari, Italy; franco.nigro@uniba.it

**Keywords:** xylem-inhabiting fungi, plant stress, nuclear magnetic resonance (NMR), hyperspectral reflectance (HSR), metabolomics, chemometrics, olive quick decline syndrome (OQDS), fingerprint, non-targeted

## Abstract

Olive quick decline syndrome (OQDS) is a disease that has been seriously affecting olive trees in southern Italy since around 2009. During the disease, caused by *Xylella fastidiosa* subsp. *pauca* sequence type ST53 (*Xf*), the flow of water and nutrients within the trees is significantly compromised. Initially, infected trees may not show any symptoms, making early detection challenging. In this study, young artificially infected plants of the susceptible cultivar Cellina di Nardò were grown in a controlled environment and co-inoculated with additional xylem-inhabiting fungi. Asymptomatic leaves of olive plants at an early stage of infection were collected and analyzed using nuclear magnetic resonance (NMR), hyperspectral reflectance (HSR), and chemometrics. The application of a spectranomic approach contributed to shedding light on the relationship between the presence of specific hydrosoluble metabolites and the optical properties of both asymptomatic *Xf*-infected and non-infected olive leaves. Significant correlations between wavebands located in the range of 530–560 nm and 1380–1470 nm, and the following metabolites were found to be indicative of *Xf* infection: malic acid, fructose, sucrose, oleuropein derivatives, and formic acid. This information is the key to the development of HSR-based sensors capable of early detection of *Xf* infections in olive trees.

## 1. Introduction

Olive quick decline syndrome (OQDS) is a disease that started to develop in the Salento peninsula (Apulia, southern Italy) in the early 2000s and subsequently rapidly spread throughout the region, causing millions of trees to die with major losses in olive groves and landscapes [1]. The causal agent of the disease was identified as *Xylella fastidiosa* subsp. *pauca*, sequence type ST53 (*Xf*), which is known as the “De Donno” strain [2,3]. This was the first report of an outbreak of this detrimental plant pathogen in the European continent and the Mediterranean Basin [4], where the pathogen is regulated as a quarantine pest, and more recently included in the list of the top 20 priority pests for the EU. As regulated pests, mandatory actions are in place to limit the expansion of the outbreaks or the epidemics currently affecting several European and Mediterranean countries, as well as to avoid further inadvertent introductions. At the European and Mediterranean Plant Protection Organization (EPPO), such a strain was then transferred from the EPPO A1 list (the list of pests recommended for regulation as quarantine pests that are absent from the EPPO region) to the EPPO A2 list (pests that are locally present in the EPPO region).

So far, about 11 million olive trees over an area of about 50,000 ha have succumbed to the infection, with significant losses for the olive and oil industry in the Apulia region [5] and an estimated economic impact of more than EUR 5 billion over the next 50 years [6].

The tolerance of olive cultivars to *Xf* has been correlated with the ability of the host plant to reduce the bacterium colonization as well as dysbiosis [7]. Among the olive cultivars, Cellina di Nardò was found to be one of the most susceptible to *Xf*, with infections leading rapidly to severe phenomena of OQDS [8,9]. *Xf* host colonization mainly affects the water movement within the plant by hindering the transporting of nutrients and signals through the xylem vessels [5]. Because *Xf* is notoriously a slow-growing bacterium, upon infection, trees often remain asymptomatic for years, hampering the detection of the disease in infected plants. Following the onset of the symptoms (wilting, shoot dieback, and desiccation), a rapid decline occurs in the plants, as a consequence of the widespread colonization in the xylem system. So far, the main strategy adopted to control the spread of infection is to eradicate infected plants when they are symptomatic. [10]. Hence, the efficient early detection of *Xf* infection would offer an advantage against the spread of the disease, enabling the timely implementation of preventive actions to protect olive growing and the related agroeconomic sector.

Over the course of the disease, from infection to the decline of the plant, the olive tree undergoes fluctuations in its morphological traits and metabolic profile. Thus, studying the metabolome changes in infected plants may offer the opportunity to develop suitable analytical methods for the early detection of the disease. Metabolomics aims to determine the metabolic profile of a sample by defining the chemical composition of small molecules (<1500 Da) [11]. In the context of spectroscopy-based metabolomics, nuclear magnetic resonance (NMR) offers a non-destructive strategy to detect metabolic fluctuations due to different stages of the disease [12,13]. Thus, a range of detectable chemical compounds could then be identified, and, finally, possible OQDS-associated metabolites could be exploited as biomarkers for the early detection of the disease [14]. Plant diseases generally show symptoms that are the combination of certain physiological and morphological alterations of the host plants. At the very early stage of the disease, visual alterations are rarely observed, and host plants appear asymptomatic. Leaf spectroscopy is one of the techniques able to display physiological, biochemical, and anatomical changes that occur in tissues as a result of such infections. Consequently, the spectral reflectance profile of both asymptomatic and healthy trees would be very similar. Nevertheless, HSR could offer a technical advantage by capturing very narrow bands of spectral reflectance (1 nm) as well as capturing the reflectance beyond the range of 400–800 nm of visible light, thus allowing for the possible early detection of plant diseases. Therefore, hyperspectral reflectance (HSR) offers a non-destructive way to record electromagnetic waves reflected by the plant and to identify specific spectral bands that can be used as signatures to detect infections before the development of symptoms [15]. Spectral signatures are related to a series of chemicals in plants, and the specificity of plant chemical fingerprints is reported to be strongly influenced by several factors [16,17]. On that foundation, the combined use of spectroscopy with physiochemistry and taxonomy is defined as spectranomics [18,19,20]. Recently, spectranomics was applied to link leaf secondary metabolites and spectral reflectance to a diverse genus of Amazonian trees [21], as well as for the early detection of Rapid ‘Ōhiʻa Death (ROD) using repeat laser-guided imaging spectroscopy (LGIS) with different derived foliar trait indices to identify susceptible trees [22].

Progress has been made in our laboratory to employ the non-targeted NMR approach [23,24,25,26,27] to reveal *Xf* infection at different levels of severity in cv. Cellina di Nardò olive trees. As a consequence of the infection, some crucial variations in the metabolic composition of foliar tissue have been observed, particularly in the signals related to malic acid, formic acid, mannitol, sucrose, and oleuropein [28].

In continuation with these preliminary studies applied on symptomatic leaves infected with *Xf*, we explored the possibility of detecting the infection in leaf tissues collected exclusively from plants that had been artificially infected with *Xf* in the initial stage of the infection process, i.e., when no symptoms can be observed during a visual inspection. To achieve this challenging task, a combined analytical strategy was explored, based on the successful results achieved in previous studies on grape and pear leaves by analyzing spectral data generated by different techniques [29,30]. Young trees of the *Xf*-susceptible cv. Cellina di Nardò were cultivated for 5 years in a temperature-controlled environment, artificially infected with *Xf*, and co-inoculated with other xylem fungi that were initially reported to be involved in OQDS [31,32]. Information derived from 1D ^1^H NMR, hyperspectral reflectance (HSR), and chemometrics were combined to select specific wavelengths in the HSR spectrum correlated with metabolites which were specifically identified as biomarkers of the *Xf* infection through the application of a chemometric study of the NMR spectral data. In this context, the identification of wavelengths specifically related to metabolites indicative of *Xf* infection in asymptomatic olive plants is a keystone for the development of proximal and remote sensing devices capable of the early detection of *Xf* infection in olive trees.

## 2. Results

### 2.1. Selection of Asymptomatic Leaves and qPCR Assay for Diagnosis of Xylella fastidiosa subsp. pauca ST53

A total number of 280 leaves were sampled from *Xf*-inoculated plants and non-inoculated plants, as summarized in Table 1. Each of the 280 leaves was subjected to HSR analysis.

The 146 leaves collected from the *Xf*-inoculated plants were subjected first to HSR analysis and then to qPCR tests to check for the presence of bacterial cells within the plant. The qPCR results showed that 69 leaves were positive for *Xf* infection, whereas the remaining 77 leaves were negative. Concerning the NMR analysis, to obtain the sample amount suitable for the extraction procedure, the leaves were arranged in groups composed of about five members each. The NMR samples derived from the *Xf*-inoculated plants were composed in such a way that the group contained at least one leaf positive for *Xf* infection according to the qPCR test. A total number of 55 NMR samples were obtained, of which 27 were from non-inoculated plants and 28 from inoculated ones (Table 1).

### 2.2. Metabolic Profile from NMR Spectral Analysis

The aqueous extracts of the olive leaves were subjected to a 1D ^1^H NOESY (nuclear Overhauser effect spectroscopy) analysis to obtain insights into the main changes in the profile of hydrosoluble compounds during the earliest phase of *Xf* infection.

**Figure 1:**
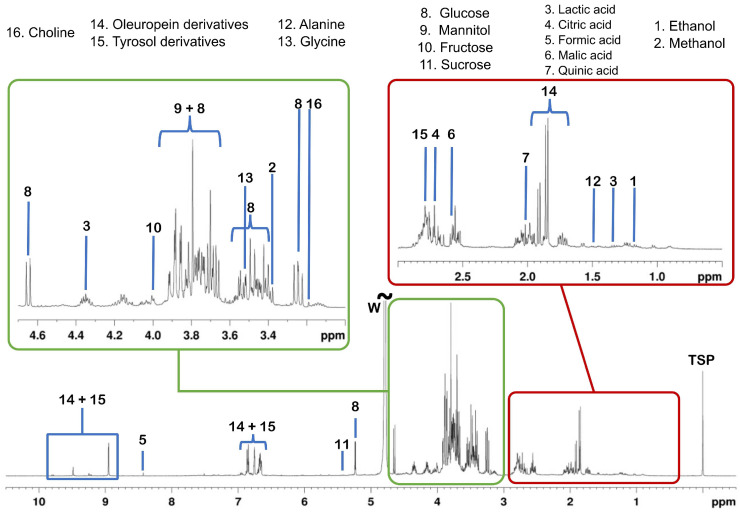
A typical 1D ^1^H NOESY spectrum of an aqueous extract of an olive leaf sample. The main classes of metabolites identified via comparison with reference compounds are indicated by increasing numbering. The full chemical shift assignment is reported in Table 2. “W” refers to the residual water signal. The chemical shift scale is referenced to the TSP-*d*_4_ singlet at 0 ppm. The metabolites’ assignment is in agreement with data reported in the literature [28,35].

The NMR spectra were analyzed through a non-targeted approach. In principle, this approach does not consider a comprehensive identification of predefined metabolites but rather aims to obtain accurate metabolic fingerprints [33,34]. In a subsequent step, it considers the overall changes in the metabolic profile and allows for biomarker discovery. The main classes of water-soluble metabolites were identified via a comparison with reference compounds. A typical 1D ^1^H NOESY spectrum of the aqueous extract of the olive leaf is shown in Figure 1.

As listed in Table 2, the main classes of metabolites detected in the samples under investigation include alcohols (methanol and ethanol), organic acids (lactic, citric, formic, malic, and quinic acids), carbohydrates (sucrose, glucose, fructose, and mannitol), amino acids (alanine and glycine), phenolic (oleuropein and tyrosol derivatives), and quaternary ammonium compounds (choline).

### 2.3. HSR Analysis

**Figure 2:**
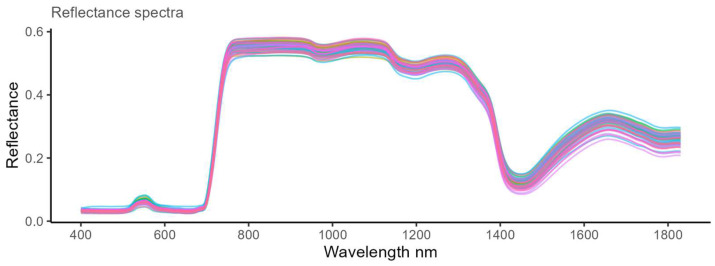
Hyperspectral reflectance (HSR) spectra of the analyzed samples with a wavelength in the range of 400–1830 nm and with a spectral resolution of 1 nm (interpolated). For each sample, the spectrum is indicated with a different color.

The HSR spectra obtained from both the *Xf*-infected and non-infected leaves had an overall similar spectral shape. Contrarily to typical symptoms of a severe stage of OQDS, asymptomatic leaves show no apparent morphological alterations neither in the color nor in the water content, as appears in the visual (400–800) and NIR (800–1830) wavelength regions, respectively. On the one hand, the spectral reflection peaks for the olive leaves were located at five regions of 560 nm, 850 nm, 1150 nm, 1280 nm, and 1650 nm. On the other hand, the spectral absorption valleys were located at five regions of 400–520 nm, 590–690 nm, 950–1050 nm, 1150–1250 nm, and 1350–1650 nm. The red edge of 700–780 nm had a positive slope (Figure 2).

### 2.4. Chemometric Analysis of NMR Data

**Figure 3:**
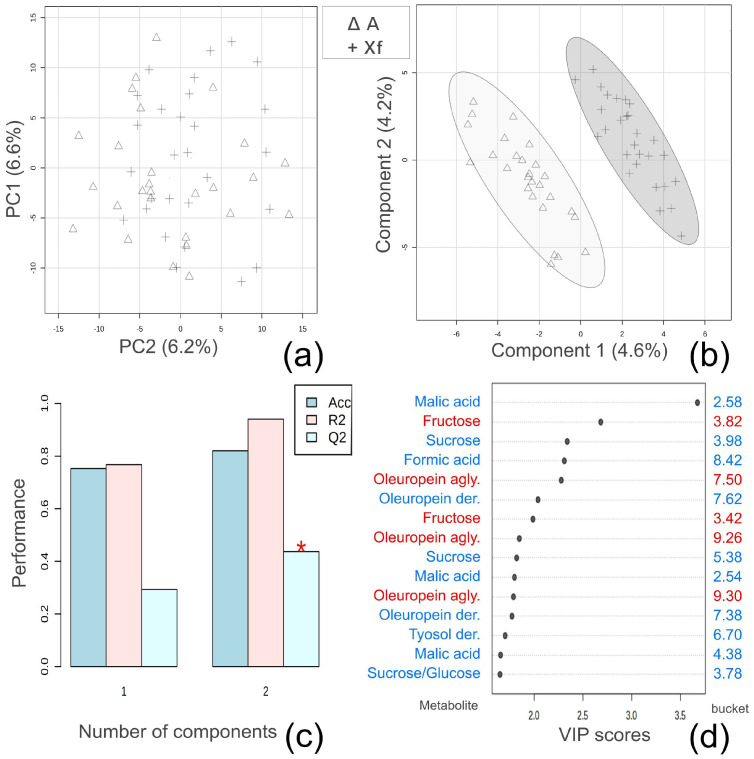
Multivariate data analysis of the NMR data in relation to *Xf* infection. *Xf*: samples from plants infected indicated with +; A: samples from non-infected plants indicated with ∆. (**a**) Scores plot obtained from the PCA showing the first two components, PC1 and PC2; (**b**) scores plot obtained from the partial least squares discriminant analysis (PLS-DA). (**c**) Ten-fold cross-validation of the PLS-DA model; Acc = accuracy. The red star indicates the component with the highest value of accumulative Q2. (**d**) Variable importance in projection (VIP) plot derived from the PLS-DA. The relative concentrations of the metabolites in each belonging class are indicated in blue and red colors for leaf extracts from *X. fastidiosa*-infected plants (*Xf*) and non-infected ones (A), respectively.

A preliminary principal component analysis (PCA) was carried out on the NMR spectral data obtained from the 1D ^1^H NOESY measurements of the aqueous extracts of the 55 NMR samples to frame the distribution of the samples with no prior knowledge of their belonging class. The samples were distributed mainly along the first two components, PC1 and PC2, accounting for 7.3% and 5.6% of the variance, respectively (Figure 3a).

To gain information about possible variations in metabolic composition related to *Xf* infection, a PLS-DA was applied to the NMR spectral data. A clear separation of the samples into two main groups was observed in the scores plot (Figure 3b). The generated PLS-DA model was validated by a 10-fold cross-validation method, which computed the following values for the first component: accuracy (0.77), R^2^ (0.77), and Q^2^ (0.28). The best values were computed for the second component with an accuracy of 0.89, R^2^ of 1.00, and Q^2^ of 0.49 (Figure 3c).

The predictive variable importance in the projection (VIP) plot (Figure 3d) was inspected to identify the first 15 variables, i.e., the buckets of the NMR spectra, which contributed more predominantly to the observed clustering of the *Xf*-infected (*Xf*) and non-infected (A) samples in the PLS-DA score plot along Component 1 (Figure 3b). The metabolites contained in the selected spectral regions were identified as depicted in Figure 3d. An analysis of the VIP graph revealed that samples obtained from uninfected plants showed relatively higher levels than the infected counterpart of the following metabolites: sugars, such as sucrose and glucose; organic acids, such as malic and formic acids; and phenolic compounds, including derivatives of tyrosol and oleuropein.

#### Correlation of NMR Diagnostics Signals to HSR

**Figure 4:**
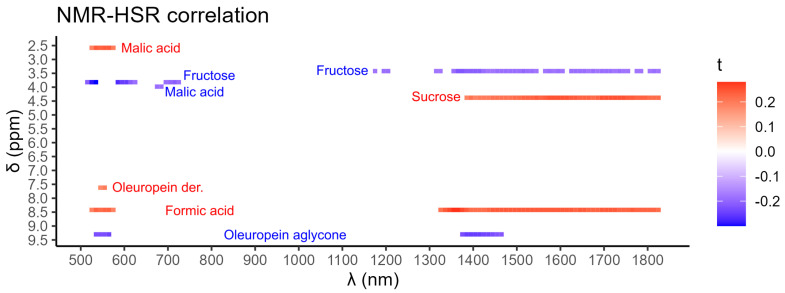
Significant correlations between hyperspectral reflectance (HSR; 10 nm wavebands; *x*-axis) wavebands and diagnostic NMR signals (0.04 ppm buckets; *y*-axis) related to *Xylella fastidiosa* infection. A positive correlation is indicated in red while a negative correlation is in blue. Kendall’s τ correlation coefficient indicated the degree of the relationship, while the *p*-value indicated the significance of such a relationship.

To identify the NMR signals correlating to HSR wavelengths concerning *Xf* infection, the 15 NMR signals reported in Figure 1 were correlated to the corresponding HSR spectra of 10 nm averaged intervals (wavebands). Significant correlations were found for the diagnostic NMR buckets of 2.58 and 4.38 ppm (malic acid), 3.42 and 3.82 ppm (fructose), 3.98 ppm (sucrose), 7.62 ppm (oleuropein derivatives), 8.42 ppm (formic acid), and 9.30 ppm (oleuropein aglycone), which in turn corresponded to wavelengths at both the visual and near-infrared regions of 510–630 nm (12 wavebands), 670–730 nm (six wavebands), 1170–1180 nm (one waveband), 1190–1210 nm (two wavebands), and 1310–1830 nm (52 wavebands). Interestingly, the wavebands located in the ranges of 530–560 nm and 1380–1470 nm had the highest amount of overlapping NMR correlations (Figure 4).

## 3. Discussion

A spectranomic study of *Xf* infection in olive trees in the very early stage of infection was attempted by searching for the relationship between the foliar spectral reflectance and the foliar metabolic profile.

In the first part of this study, the variations at the metabolic level of aqueous extracts of leaves collected from *Xf*-infected plants versus non-infected ones were deeply investigated. Such a metabolomic study was performed through a non-targeted NMR-based approach, following which a decrease in malic acid, sucrose, formic acid, oleuropein derivatives, tyrosol derivatives, and glucose was observed in the infected leaves. The same samples showed an increase in fructose and oleuropein aglycone. Such variations at the metabolic level are reported as a result of the plant–microbe interaction [36,37,38]. This is manifested as a parasitic symbiosis between *Xf* and the susceptible olive cultivar, Cellina di Nardò, where the bacterium benefits while the olive tree suffers harmful outcomes [39]. In that case, the plant metabolome succumbs to a breakdown by enzymes produced by *Xf* that serve to degrade the pit membrane of the plant xylem, which leads to impairments in plant physiological properties such as photosynthesis [40].

Oleuropein represents the main phenolic secoiridoid metabolite present in olive leaves and fruit [41]. A pool of molecules is reported to originate from oleuropein, from which are the aglycone form of oleuropein, the secoiridoid compounds derived from the elenolic acid ring opening with different terminal rearrangements, the aldehydic forms of elenolic acid, and some phenolic compounds [42], such as hydroxytyrosol and tyrosol [43]. The different derivatives of oleuropein were rather decreased in extracts of the leaves collected from the *Xf*-infected plants. Interestingly, the aqueous leaf extracts collected from the *Xf*-infected plants were characterized by a higher content of oleuropein in the aglycone and fructose form. Such compounds may arise from the breakdown of glycosidic bonds within oleuropein and sucrose, respectively.

Furthermore, other phenolic metabolites that are typically present in olive leaves, including tyrosol derivatives, were also found to be less abundant in *Xf*-infected plants. The lower content of phenolic derivatives in the leaves of plants inoculated with *Xf* could be explained by the fact that, as reported in the literature, phenolic compounds are particularly sensitive to biotic and abiotic stress conditions. Phenolic compounds, such as flavonoids, secoiridoids, and hydroxycinnamic acid derivatives, act as antioxidants and are involved in plant defense by counteracting plant oxidative stress [44,45,46,47]. On the other hand, the aqueous extracts of leaves harvested from the *Xf*-infected plants showed a relatively higher content of oleuropein aglycone derivatives. Such compounds are most likely produced during the metabolism of oleuropein [48].

Fructose, glucose, and sucrose were also reported to play an important role in *Xf* infection. Coherently to the data reported in the literature, a relatively higher amount of fructose was associated with *Xf* infection. Moreover, a decrease in sucrose and glucose contents was also reported in *Xf* infection. On the other side, an increase in fructose was observed in other studies reported in the literature related to the *Xf* infection of olive trees, where it was observed that, regardless of the cultivar under investigation, the levels of this sugar were higher in the infected plants [49]. The levels of sugars in plant cells as well as their transport, utilization, and storage are regulated and strongly dependent on cell physiological activity, and thus directly correlate to the plant response to stress, including pathogen infection. Numerous studies have also shown that sugars play a key role in plant defense responses to various abiotic and biotic stress factors [50,51]. It is well documented that sugars are not only the main substrates utilized in respiration processes, supplying energy for cellular defense responses against pathogens but also provide the carbon source for the synthesis of defense compounds [52,53]. In addition, sugars represent metabolic signaling molecules in host plant cells, which induce the expression of many defense genes [54,55]. The observed decrease in sucrose may be due to a degradation process of sucrose to fructose and glucose by enzymes, such as β-fructafuranosidase. The relatively lower content of glucose in aqueous leaf extracts from *Xf*-infected plants compared to that from non-infected plants may be explained by the fact that glucose, in addition to being a universal source of carbon, also acts as a signaling molecule by modulating various metabolic processes in plants. Glucose is involved in the regulation of the production of antioxidants and compounds similar to those of the photosynthetic fixation of CO_2_, which act as osmoprotectants by reducing membrane permeability during stress [56]. The higher amount of fructose relative to *Xf* infection was suggested to not be directly involved in the osmoprotection that generally occurs in the plant during infection [51]. Rather, this might be simply due to the hydrolysis of sucrose into glucose and fructose by *Xf*, with fructose not being the preferred carbon source for *Xf* when sucrose and glucose are present [57,58].

Furthermore, a lower malic acid content was observed in the infected samples compared to the non-infected counterparts. Malic acid is a key metabolite in the biosynthesis of amino acids and fatty acids and is involved in the citric acid cycle [59]. In a previous NMR-based metabolomics study, a decrease in malate content was observed in symptomatic Huanglongbing (HLB) leaf extracts [60]. The levels of this metabolite in plant cells have been reported to undergo significant variations under physical (cold, heat, drought), chemical (herbicides, pesticides, pollutants), and pathogenic stress [61,62]. Malic acid levels are often associated with the activity of the NADP-malic enzyme (NADP-ME), responsible for the decarboxylation of this organic acid with the production of pyruvic acid [63].

In the second stage of this study, the HSR spectral data were correlated to the fifteen most significant NMR spectral regions according to the VIP plot of the PLS-DA model. Since the spectral reflectance data, such as HSR, contains overlapping signals from both the chemical composition of the sample and physical effects, such as light scattering, as well as artifacts such as illumination/shadows [64], it is usually necessary to separate the physical information from chemical information [65,66]. However, to model the additive/multiplicative effects in the sample, it is required to use the whole spectrum, which is not feasible for spectral data acquired using instruments such as drones, which are typically equipped with few wavebands, usually around six to twelve channels [67]. Furthermore, some studies reported the early detection of plant diseases using HSR [22,68,69]. Nevertheless, such studies did not unmix the overlapping signals in the spectral reflectance data. In this study, the HSR was also not unmixed but was rather derived from the NMR signals that are diagnostic for *Xf*. Thus, the HSR is only selected based on a chemical (metabolic) fingerprint.

As a result, strong correlations were found between some wavebands of the HSR spectra and the following metabolites: oleuropein derivatives, oleuropein aglycone, sucrose, fructose, and malic acid. Specifically, the wavebands located in the ranges of 530–560 nm and 1380–1470 nm showed the highest amount of overlapping NMR correlations.

The significantly correlating wavebands found in this study were in agreement with results found in a recent study, in which a PLS regression model was applied for predicting the DNA content of *Xf* in leaves using HSR [40]. In that study, four wavebands at ranges of 360–520 nm, 550–620 nm, 690–860 nm, and 1390–1490 nm had the highest VIP score for predicting the DNA content. All these ranges, except the first one, were correlated for the diagnostic NMR signals, which is expected to be similar, since, in that study, the HSR data were pre-processed via multiplicative scatter correction (MSC), which removes additive and multiplicative components that result from physical effects, e.g., light scattering. Nevertheless, the use of MSC on HSR cannot extend to reduce the number of wavebands for use in an instrument equipped with few waveband channels, since MSC depends on the use of the whole spectrum [70,71].

Malic acid, as well as oleuropein derivatives, were significantly positively correlated to the green color waveband at around 550 nm. It is reported in the literature that the wavebands at 500–700 nm are related to plant pigments, which are in turn related to photosynthesis and chlorophyll activity [69]. Such a waveband was beneficial in differentiating between healthy tomato leaves and those with tomato yellow leaf curl disease caused by *tomato yellow leaf curl virus* (TYCLV) [72]. Furthermore, this waveband was also exploited to estimate moisture content in Miscanthus along with other wavebands in a MicaSense RedEdge-Mx multispectral camera [73].

The waveband at around 1400 nm is associated with water absorption, which interestingly plays a major role in OQDS [74]. In the xylem vessels, *Xf* grows and multiplies by forming a biofilm that, over time, hinders the regular water movement through the xylem vessels [75]. Moreover, this waveband was also associated with diagnostic NMR signals for pear leaves infected with *Erwinia amylovora* [29]. Specifically, the wavebands at 1391 nm and 1455 nm were found to be indicative of water stress in olive leaves [76]. On the one hand, these wavelength regions were found to be significantly negatively correlated to both fructose and oleuropein aglycone, which indicates a higher absorbance for those wavebands. On the other hand, they were significantly positively correlated to sucrose, denoting lower absorbance for those wavebands by *Xf*-infected leaves.

In conclusion, the feasibility of a spectranomic approach to reveal OQDS-related diagnostic wavebands at the very early stages of the infection process of olive leaves with *Xylella fastidiosa* subsp. *pauca* ST5 (*Xf*) was explored. Hyperspectral reflectance wavebands (average 10 nm spectral ranges) were correlated with NMR spectral regions (0.04 ppm buckets) which, based on PLS-DA, were found to be diagnostic of the presence of *Xf* infection.

Both positive and negative correlations were obtained between specific band waves, mainly in the 510–630 nm and 1310–1830 nm regions, with a pool of metabolites, including fructose, sucrose, malic acid, and oleuropein derivatives. The information collected during this study paves the way for the development of HSR-based sensors, which can be appropriately adapted to selectively reveal even subtle optical changes on the surface of asymptomatic infected leaves, enabling the early detection of *Xf* infection in olive trees.

## 4. Materials and Methods

### 4.1. Cultivation of Bacteria and Fungi

A suspension was prepared from the “De Donno” strain (database name: CIRM-CFBP French Collection for Plant Associated Bacteria; accession number: CFBP 8402) of *Xf* [77] taken from an 8–10-day old buffered charcoal yeast extract agar (BCYE) culture medium grown at 28 °C, resuspended in a phosphate-buffered saline solution (PBS, 0.05 M, pH 7.2), and adjusted to 0.5–0.6 absorbance at an optical density of 600 (OD600), which corresponds to a concentration of 10^9^ colony forming units (CFUs)/mL [78]. A sterile PBS solution was used as a control.

The employed mycelial plugs (4 mm) consisted of five selected fungal isolates, i.e., *Phaeoacremonium aleophilum* (B1a), *Phaeoacremonium rubrigenum* (N20), *Pseudophaeomoniella oleae* (Fv84), *Pseudophaeomoniella oleicola* (M24), and *Pseudophaeomoniella oleicola* (M51). The mycelial plugs of each fungal isolate were obtained from two-week-old potato dextrose agar (PDA) culture media grown at 23 °C. The sterile PDA plugs were used as a control.

### 4.2. Cultivation and Artificial Infection of the Olive Plants

Olive plants (*Olea europaea* L. cv. Cellina di Nardò) were cultivated in a quarantined greenhouse under a controlled environment of 23–24 °C in winter and 25–30 °C in summer under >80% relative humidity at the CNR research area of Bari (Italy). After two years, the plants were inoculated with *Xf* and fungi according to a reported procedure [4]. Table 3 summarizes the adopted experimental design.

Artificial infection with *Xf* was performed via pinprick inoculation [4,79,80,81]. Specifically, aliquots of 10 µL of the PBS suspension containing *Xf*, prepared according to the procedure described above, were punched with sterile entomological pins 5–6 times. For each plant, the 9–12 inoculation sites were carried out on three consecutive leaf nodes of 3 to 4 twigs placed 40–50 cm from the ground.

Four weeks after the artificial infection with *Xf*, the fungal isolates and the PDA samples were inoculated. To perform the inoculation, the bark of the main trunk at 40–50 cm from the soil was cut (5 mm area in diameter) and removed using a sterile cork-borer. The removed bark was replaced with the mycelial plug or the PDA in the case of the control sample. Then, sterile wet cotton was wrapped around the inoculation site and further wrapped with Parafilm to ensure normal hydration.

All sampled leaves were visually inspected for OQDS symptoms, assuring that all of them belonged to the severity levels of 0 and 1, which were regarded as asymptomatic/having early symptoms of OQDS, respectively [8].

### 4.3. Diagnosis of Xylella fastidiosa subsp. pauca ST5 (Xf) in Inoculated Plants using qPCR Assay

DNA was extracted from leaves using a CTAB (cetyltrimethylammonium bromide)-based method [82]. Pieces of midveins and petioles (ca. 0.5 g) were hammer-smashed in sterilized plastic bags. Then, 5 mL of CTAB buffer (2%, 0.1 M Tris-HCl pH 8, 20 mM EDTA, and 1.4 M NaCl) was added, followed by homogenization through a Homex 6 homogenizer (Bioreba, Reinach, Switzerland). Aliquots of the resulting homogenized suspension (1 mL) were transferred into 2 mL microcentrifuge tubes containing 1 mL of chloroform, followed by incubation at 65 °C for 30 min using a water bath. Finally, the aqueous phase was separated and treated with 0.7 volumes of cold isopropanol, inducing the DNA precipitation, which was, subsequently, used for qPCR analysis [83].

Quantitative real-time polymerase chain reactions (qPCRs) were performed using 20 µL reaction volumes containing 10 µL of 2 X qPCR Supermix-UDG (Invitrogen; Thermo Fisher Scientific Inc., Waltham, MA, USA), reaching a final concentration of 4 mM of MgCl_2_, 300 nM of *Xf* forward *XF*-F: 5′-CACGGCTGGTAACGGAAGA-3′ and reverse *XF*-R: 5′-GGGTTGCGTGGTGAAATCAAG-3′ primers, 100 nM of dual-labeled fluorescent probe *XF*-P: 5′-TCGCATCCCGTGGCTCAGTCC-3′ labeled with 5′-Fluorescein/Black Hole Quencher 1 (6-FAM/BHQ-1), bovine serum albumin (BSA) at 300 ng/µL (Sigma-Aldrich, Milan, Italy), and 2 µL of the DNA template [84].

The thermocycling conditions were 50 °C for 2 min, 94 °C for 2 min, then 40 cycles of 94 °C for 10 s and 62 °C for 40 s. All samples were amplified in triplicates. Threshold values were applied automatically through the CFX Manager V1.6 software (Bio-Rad Laboratories, Hercules, CA, USA).

### 4.4. Hyperspectral Reflectance (HSR)

HSR was acquired through a hyperspectral acquisition system. This system consisted of a FieldSpec^®^3 spectroradiometer (Analytical Spectral Device (ASD), Boulder, CO, USA) linked via an optical fiber cable to a leaf probe (ASD) and a leaf clip holder (ASD) in addition to an instrument controller (laptop) to display and save the data. The plant probe was of a 10 mm spot size, with internal illumination from a halogen bulb of 2901 K ± 10% color temperature. The HSR data were in the range of 350–1830 nm, with spectral sampling intervals of 1.4 nm and 2 nm at spectral ranges of 350–1050 nm (full width at half maximum, FWHM: 3 nm) and 1000–1830 nm (FWHM: 10 nm), respectively.

The acquisition system (plant probe, optical fiber, and spectroradiometer) was fixed to a firm workbench to avoid noise generation due to optical fiber motion during acquisition. The leaves were collected from plants and the upper surface was subjected to HSR analysis. The raw spectral signatures acquired through the RS^3^™ software v.3 (ASD) were pre-processed using the ViewSpec Pro 6.0.10 software (ASD). To acquire high-quality spectra, the spectroradiometer was turned on and left heating for 45 min, and the optical fiber cable was immobilized using sticky tape to prevent noises caused by its movement. Moreover, for each measurement, the device performed an automatic procedure of calibration and light optimization using a white reference (Spectralon, Labsphere, North Sutton, NH, USA). Next, the leaf clip of a 10 mm spot size was placed in the middle of the leaf, beside the midrib, to collect uniform measurements, and the integration time was set to 68 ms during measurements. Eight to ten leaves from each studied plant were used for hyperspectral data acquisition, and in each observed leaf, five reflectance measurements were taken and averaged.

The final resolution of 1 nm was obtained via the subsampling and interpolation of the spectral channels and recorded as relative reflectance values using the RS^3^™ software v.3 (ASD). All the acquired raw HSR spectra were stored in a “.asd” format, while the pre-processed raw data were stored in a “.ref” format and then exported in a “.txt” format (American Standard Code for Information Interchange, ASCII) and imported in a MATLAB R2021a routine (The MathWorks Inc., Natick, MA, USA) developed by the authors for further filtering. First, a Savitzky–Golay filter [85,86] with a frame size of 15 data points (second-degree polynomial) was applied. Then, the UV region from 350 nm to 400 nm was truncated, and the interval ranging between 400 nm and 1830 nm was considered for further analysis. The pre-processed spectra were exported in comma-separated values in a “.csv” format.

### 4.5. NMR Sample Preparation and Spectra Acquisition

For the NMR sample preparation, 3-(trimethylsilyl)-2,2,3,3-tetradeutero-propionic acid, sodium salt (TSP-*d*_4_, CAS N. 24493-21-8, 99% D, Armar Chemicals, Döttingen, Switzerland), hydrochloric acid (HCl, 37%, CAS N. 7647-01-0; ≥99.5%, Sigma-Aldrich, Milan, Italy), sodium oxalate (Na_2_C_2_O_4_, CAS N. 62-76-0; ≥99.5%, Sigma-Aldrich, Milan, Italy), sodium azide (NaN_3_, CAS N. 26628-22-8; ≥99.5%, Sigma-Aldrich, Milan, Italy), deuterium oxide (D_2_O, CAS. N. 7789-20-0, 99.86% D, Eurisotop, Saclay, France), and 509-UP 7 NMR tubes (Norell, Landisville, NJ, USA) were used.

After HSR acquisition, the collected olive leaves were lyophilized at 223 K under 0.180 mbar for 72 h in a Christ Alpha 1–4 LSC lyophilizer (Martin-Christ Gefriertrocknungsanlagen GmbH, Osterode am Harz, Germany). The dried samples were then ground in a blender, sieved through a mesh of 0.5 mm pores, and stored at 253 K. For the preparation of each NMR sample, an amount of 50 mg of olive leaf powder and 1.5 mL of oxalate buffer (Na_2_C_2_O_4_ (0.25 M), NaN_3_ (2.5 mM)) at pH 4.2 (adjusted via the addition of HCl (37%)) were mixed and sonicated at 40 kHz for 10 min. Next, the samples were vortexed at 2500 rpm for 5 min (Advanced Vortex Mixer ZX3, VELP Scientifica Srl, Usmate, Italy), then centrifuged at 4700× *g* for 15 min (ROTOFIX 32 A, Hettich, Italy). After centrifugation, an automated system for liquid handling (SamplePro Tube, Bruker BioSpin GmbH, Rheinstetten, Germany) transferred 630 μL of the supernatant solutions into NMR tubes containing 70 μL of 0.20% of sodium salt of the 3-trimethylsilyl-2,2,3,3-tetradeuteropropionic acid (TSP-*d*_4_) solution in D_2_O.

The 1D ^1^H NOESY spectra were recorded using a Bruker Avance I 400 MHz spectrometer equipped with an autosampler and a 5 mm inverse probe (Bruker BioSpin GmbH). The 1D ^1^H NOESY spectra were acquired using the pulse program (noesygppr1d). The following parameters were applied: number of scans: 128; data points: 64 K; spectral width: 8013 Hz; 90° pulse angle: 8.16 μs; acquisition time: 4.09 s; mixing time: 10 ms; recycle delay: 6 s. Each spectrum was acquired using the Topspin 2.1 software (Bruker BioSpin GmbH) under an automatic process that lasted around 22 min and included sample loading, temperature stabilization for 5 min at 298.2 K, tuning, matching, and shimming. The NMR raw data (Free Induction Decays, FIDs) were processed using the software MestReNova 11.0 (Mestrelab Research SL, Santiago de Compostela, Spain). The FIDs were zero-filled to 128 K number of points and then underwent a Fourier transformation through the application of an exponential multiplication function with a line broadening of 0.1 Hz. The phase and baseline were automatically corrected, and the TSP-*d*_4_ singlet signal set at δ = 0.00 ppm was used as a chemical shift reference.

### 4.6. Chemometric Analysis

The 1D ^1^H NOESY spectra related to the 55 aqueous extracts of olive leaves were segmented into buckets of 0.04 ppm intervals in the range of [10, 0.50] ppm using MestReNova 11.0 (Mestrelab Research SL, Santiago de Compostela, Spain). The underlying area of each bucket was normalized to the total intensity. The buckets in the region [5.10, 4.60] ppm, corresponding to the residual water signal, were set to 0.

The obtained data matrix was imported into MetaboAnalyst 5.0 [87], and buckets were subjected to mean-centering and divided by the standard deviation of each variable (unit variance scaling). The NMR spectra constituted the observations, while the buckets constituted the x-variables. Initially, the unsupervised method of principal component analysis (PCA) was performed to obtain an overview of the data. Then, the supervised partial least square–discriminant analysis (PLS-DA) was used as a supervised method that uses multivariate regression techniques to extract the information that can predict the class membership (Y) via a linear combination of original variables (X). The two classes, namely *Xf*-infected (*Xf*) and non-infected (A), counted for 27 and 28 observations, respectively.

The performance of the PLS-DA model was evaluated based on the R^2^ (goodness-of-fit) and Q^2^ (goodness-of-prediction) parameters. Q^2^ is an estimate of the predictive ability of the model and is calculated via 10-fold cross-validation (CV). In each CV, the predicted data are compared with the original data, and the sum of squared errors is calculated. Good predictions will have a high Q^2^ [88].

The relationship between the diagnostic NMR signals and HSR spectra was determined using a correlation-based approach [89,90]. To obtain a homogenous sample size between both techniques, the 280 HSR spectra were conformed to the 55 NMR samples by taking the median of each corresponding group of leaves, achieving ca. five HSR spectra for each NMR spectrum. The HSR and the NMR data were correlated using Kendall’s correlation. This non-parametric correlation method was chosen based on the mechanistic representation of the nature of the NMR signals and HSR wavebands. While the 1D ^1^H NOESY spectra linearly correspond to the concentration of the metabolites in the sample [91], the HSR spectra respond on a non-linear scale to the chemical effects of light absorption (involving photochemical and thermal effects) at various wavelengths [92]. In such circumstances, in principle, measuring the ranks between the HSR and NMR data through Kendall’s correlation would be more suitable than using a product-moment-based correlation such as Pearson’s correlation test. Furthermore, Kendall’s correlation method was chosen rather than Spearman’s one as the former is generally less prone to biases caused by outliers and/or slight variations in the variables under investigation.

It was assumed that if there was an association between the NMR and HSR profiles, the correlation matrix would show a strong negative or positive correlation (slope of the line), with maximum values of the correlation coefficient equal to −1 and 1, respectively. On the other hand, if there was no relationship, the correlation would have values near zero [21]. Importantly, the *p*-value determines how well this slope fits the data points, where a *p*-value of <0.05 is considered to be significant. The calculation and visualization of the correlation matrices were performed in RStudio IDE of the R programming language, using core packages as well as the tidyverse metapackage [93,94,95].

## Figures and Tables

**Table 1:** List of samples subjected to the HSR and NMR measurements.

	N° Leaves	Negative	Positive	HSR Samples (One Leaf)	NMR Samples (~5 Leaves)
Non-inoculated	134	134	0	134	27
*Xf*-inoculated	146	77	69	146	28

**Table 2:** List of metabolites contained in the aqueous extracts of leaf samples and identified via 1D ^1^H NOESY measurements.

Compound ID	Compound	δ (ppm)	Multiplicity	J (Hz)
Alcohols				
1	Ethanol	1.18	t	6.5
		3.65	q	6.5
2	Methanol	3.33	s	
Organic acids				
3	Lactic acid	1.34	d	6.9
		4.15	q	6.9
4	Citric acid	2.70	d	15.0
		2.80	d	15.5
5	Formic acid	8.43	s	
6	Malic acid	2.54	dd	15.8, 8.7
		2.77	dd	15.8, 3.9
		4.35	dd	8.7, 3.9
7	Quinic acid	1.87	dd	13.4, 10.8
		1.96	m	
		2.07	m	
		3.56	dd	9.3; 3.3
		4.04	m	
		4.14	q	3.5
Carbohydrates				
8	Glucose	3.24	dd	9.2, 7.9
		3.43	m	
		3.48	m	
		3.53	dd	9.8, 3.8
		3.75	m	
		3.83	m	
		3.87	qd	11.8, 2.4
		4.65	d	7.9
		5.23	d	3.7
9	Mannitol	3.68	dd	11.6; 6.2
		3.77	m	
		3.81	d	8.6
		3.88	dd	11.6; 2.5
10	Fructose	3.57	m	
		3.71	dd overlapped	
		3.79	m overlapped	
		3.90	dd overlapped	
		4.00	m	
		4.03	m	
		4.11	m	
11	Sucrose	3.48	t	9.2
		3.57	dd	9.9; 3.7
		3.67	s	
		3.78	t	9
		3.83	m	
		3.87	m	
		3.91	dd	6.2; 3.5
		4.05	t	8.5
		4.22	d	8.7
		5.42	d	3.8
Amino Acids				
12	Alanine	1.49	d	7.3
		3.80	q	7.3
13	Glycine	3.53	s	
Phenolic compounds				
14	Oleuropein derivatives	1.85	dd (methylenic proton of derivatives)	
		1.91		
		6.67	multiplets (aromatic protons of derivatives)	
		6.79		
		7.5		
		8.95	dd (aldehydic protons of the aglycone forms)	
		9.02		
		9.20		
		9.21		
		9.25		
15	Tyrosol derivatives	2.78	t overlapped	
		3.78	t overlapped	
		6.94	m	
		6.75	m	
		7.12	m	
		7.14	m	
Quaternary ammonium compounds				
16	Choline	3.20	s	
		3.50	dd overlapped	
		4.05	m	

**Table 3:** The experimental design describing the combination of different fungal isolates with *Xf*-infected and non-infected plants.

Fungi	Isolate Code	Non-Infected Plants (A)		*Xf*-Infected Plants (*Xf*)	
		Label	N Samples	Label	N Samples
Control	-	A-A	5	*Xf*-A	7
*Phaeoacremonium aleophilum*	B1a	A-F1	4	*Xf*-F1	5
*Phaeoacremonium rubrigenum*	N20	A-F2	5	*Xf*-F2	4
*Pseudophaeomoniella oleae*	Fv84	A-F3	4	*Xf*-F3	4
*Pseudophaeomoniella oleicola*	M24	A-F4	3	*Xf*-F4	5
*Pseudophaeomoniella oleicola*	M51	A-F5	6	*Xf*-F5	3
Total		6	27	6	28

## Data Availability

Data are contained within the article.
